# King Edward's Hospital Fund

**Published:** 1922-02

**Authors:** 


### KING EDWARD'S HOSPITAL FUND.

**(From a Correspondent.)**

THE meeting of the General Council of King Edward's Hospital Fund for London at which the annual grants to hospitals were made was remarkable in two respects. For, apart from its immediate purpose, it raised the questions of the fund's capital and its new organisation of Committees. The bequest to the Fund of the residue of the estate of the late Lord Mount Stephen has already been recorded in our columns, but it is too early yet to estimate what the residue will be. Lord Mount Stephen had fostered the Fund's capital account from its foundation; and before his death had given to it over £500,000.

Since the Fund in the previous year had to draw on its reserves it is good to know that during 1921 £74,000 had been received in legacies by December. This has enabled £220,000 to be distributed, or £20,000 more than in 1920, of which the major part was earmarked for maintenance. Capital grants for extensions and improvements were reduced.

The Fund, in reply to a request, is preparing a scheme of provident contributions, in which it has invited the co-operation of the Hospital Saturday Fund. The Fund is also preparing a general scheme for a combined appeal.

These new tasks have led to a reorganisation, whereby the new numerous functions of the Fund will be allotted to Committees, the chairmen of which will meet regularly on the Committee of Management. Since January 1, the duties hitherto performed by the King's Fund Policy Committee have been transferred to this Committee of Management.

It was the Distribution Committee which recommended the Voluntary Hospitals Commission to make emergency grants to 24 hospitals in danger of closing beds, and in November the Commissioners distributed £77,900 to these 24 hospitals, though not on the same basis as that which had been adopted by the Distribution Committee.

Since, before any grants other than emergency grants are made, fresh money must be raised or in sight equal to the combined amounts of such emergency grants and further grants, the King's Fund has consulted the conference of central agencies and the London Regional Committee of the British Hospitals Association, and prepared schemes for provident contributions and for a combined appeal, which have been laid before the London Regional Committee.

The King's Fund, which acts as the Local Committee for London, is thus a model to Local Committees in the provinces, though, unlike them, it is not a new body. The report of the Distribution Committee shows, apart from the grants, how the Fund controls economy and can check waste. In dealing with the routine expenses and income of the London Hospitals since 1913 (the increases are respectively 135 and 66 per cent.), it is noted from Lord Cave's report that the aggregate increases in expenditure in London, where comparative statistics of cost of working are circulated by the King's Fund, are considerably smaller than the aggregate increases of the provincial hospitals. Further, the Distribution Committee, where necessary, calls attention privately to high cost, and in a few cases suggests that if a reduction to a figure nearer the average is not effected, it may feel compelled to recommend a public remark next year.

The Fund not only has stabilised hospital finance, but provided a continually increasing pressure in the direction of co-ordination.

---

THE authorities of the voluntary hospitals in Scotland are apprehensive of their prospects of getting a fair share of the £500,000 at the disposal of the Voluntary Hospitals Commission. It is felt that this sum will be largely swallowed by the London hospitals, and that the great teaching hospitals of Edinburgh and Glasgow particularly will receive little, if anything, more than the advice of the Local Voluntary Hospitals Committees. The Scottish authorities claim modestly, and, we think, with some justification, that it was merely their foresight in adjusting their management to the crisis that enabled the Scottish hospitals to maintain a slightly better position than the London hospitals. Sir Napier Burnett's recent analysis of the financial position of the Scottish hospitals, it will be remembered, testified to the efficiency of voluntary hospital management in Scotland.

